# Work Productivity Loss in Inflammatory Bowel Disease Patients in Turkey

**DOI:** 10.1155/2020/6979720

**Published:** 2020-01-08

**Authors:** Firdevs Topal, Hakan Camyar, Elif Saritas Yuksel, Suleyman Gunay, Fatih Topal, Emine Özlem Gür

**Affiliations:** ^1^Izmir Katip Celebi Univ Gastroenterology Department, Turkey; ^2^Izmir Katip Celebi Univ Ataturk Research and Teaching Hospital Gastroenterology Department, Turkey; ^3^Izmir Katip Celebi Univ Emergency Department, Turkey; ^4^Izmir Katip Celebi Univ Surgery Department, Turkey

## Abstract

**Background:**

Beyond the medical treatment in inflammatory bowel disease (IBD), there are other issues which influence the quality of life adversely. The aim of this study was to determine the impact of the IBD patients' illness on working and education life.

**Method:**

The participants were invited to participate in the online survey from the Turkish Crohn's and Ulcerative Colitis Patient Association network. The data was analysed and then discussed to improve the health-related quality of working and education life.

**Results:**

One hundred and fifteen patients had ulcerative colitis (UC) (57.2%), and 86 had Crohn's disease (CD) (42.8%). There was a statistically significant difference in UC between retirement age group 1 (<40 age) and groups 2 (40-49 ages) and 4 (60-65 ages) (*p* < 0.05). There was the same significant difference in CD. Even though the data did not have significant statistical difference, there was clustering around negative perceptions the patients have about their working and education lives.

**Conclusion:**

Our survey revealed a very strong causative relationship between work and IBD involving problems before, during, and at the end of employment. Young patients lower their career expectations, and that announces a clear need to support them and improve career guidance.

## 1. Introduction

Inflammatory bowel disease (IBD) is a general term for Crohn's disease (CD) and ulcerative colitis (UC). A very small group cannot be classified (unclassified IBD) (IBD-U).

In North America, incidence rates of IBD are between 2.2 and 19.2 cases per 100,000 person-years for ulcerative colitis and 3.1 and 20.2 cases per 100,000 person-years for Crohn's disease. The incidence and prevalence of IBD are low in Asia and the Middle East; nonetheless, in some newly industrialized countries in Africa, Asia, and South America, the incidence is rising [1]. In recent years, the prevalence of the disease has increased especially in eastern countries where the disease was not prevalent before even though plateauing incidence rates have been reported in northern and western regions of the world [2]. In Turkey, there are several epidemiological studies on IBD. In the beginning of the 2000s, the incidence was found to be 4.4/100,000 and 2.2/100,000 for UC and CD, respectively [3]. In a new study, it was found that mean annual incidences increased from 0.99/105 and 0.45/105 (2004 to 2005 years) to 4.87/105 and 2.09/105 (2011 to 2013 years) for ulcerative colitis and Crohn's disease, respectively, in the western Black Sea Region of Turkey [4].

On the other hand, a better understanding of the disease and newly developing drugs has expanded the life expectancy of the patients. A longer life span with a chronic disease brought out another issue which is the quality of life. Working life and education are affected by the disease. The employers might be disturbed by the patient's frequent needs to attend appointments with the clinician. Weakness, pain in both the joints and abdomen, and frequent need for the toilet can deprive the patient from work and daily life tasks. In the case of incontinence, patients can refrain from work especially if the access to a toilet is finite.

Sick leave and unemployment in the IBD population were found out to be significantly correlated with a reduction in patient-reported HRQoL (health-related quality of life) [5]. The authors hypothesized that the measured HRQoL in fact reflects the socioeconomic safety and stresses that a patient experiences. Older investigations showed a negative impact of IBD on labor participation [6]. IBD is a chronic disease, and patients with chronic diseases often present increased rates of absenteeism [7]. The absenteeism scores found in the mentioned [5] cohort were similar to those found in patients with rheumatoid arthritis (7.7 vs. 5.6% for CD and 6.5% for UC patients), indicating that chronic diseases affect patients' work capacities.

Most of the healthcare costs are related to chronic illnesses [8]. The loss of productivity from employee absenteeism is as important as the healthcare costs. Unsalaried days off from work due to illnesses and early retirement are the most usual reasons for a decrease in the income for the employee. These problems are mostly seen due to chronic illnesses between the ages of 45 and 64 [9]. Financing preventive health measures and improving the awareness of the chronic disease in both the patients and the working life aspects such as the employers and the colleagues improve the patients' well-being and welfare in conjunction with overcoming the adverse impacts that ill health has on workforce participation.

Presenteeism is another issue for IBD patients, which is defined as the act of being present at work even if one is too sick to be productive or work beyond the expected hours.

In the recent years, patient-reported outcomes (PROs) have been accepted as important endpoints in clinical trials and as treatment targets in real life. Health-related quality of life (HRQoL) is mostly known and used PRO; however, it does not focus on the working life and absenteeism and presenteeism [10].

The aim of our study was to determine the impact of the IBD patients' illness on working life. All patients were invited to fill up a survey through the Internet. Identifying which problematic issues in laboring cluster around which type of disease and which kind of demographic findings was the main objective. There was a group of young patients still studying in high school or university, and they were asked about their concerns about the rest of their education and then the upcoming employment life. After putting forward the impeding issues in patients' point of view, we planned to discuss what can be done to improve the health-related quality of working life.

## 2. Materials and Method

The study was conducted according to the Declaration of Helsinki and was approved by the institutional review board. Written informed consent was obtained from all participants.

This was a prospective, web-based, observational cohort study of patients with mild-to-moderate IBD outpatients.

The survey was designed to collect qualitative and quantitative information about the working and education life of the patients and was formed to encourage respondents to provide unbiased and definite information. The questions were designed so that participants understand the questions and are not likely to refuse to answer or distort the answers. The questions were in Turkish. It was a baseline questionnaire about health, education, employment situation, work performance, work-related problems, and perceived disability.

The inclusion criteria were as follows: aged ≥18 years at the time of enrollment and previously diagnosed with IBD with a mild-to-moderate course at the time of the survey. The disease severity of mild-to-moderate UC was defined according to the Mayo scale (6–10 points for moderate) [11]. The disease severity of mild-to-moderate CD was defined according to the CD Activity Index (CDAI) (<450 points for moderate) [12].

The survey consisted of three parts: (1) questions related to demographic features and disease type and course, (2) assessment of work disability, and (3) assessment of education disability. Some questions were answered by using the Likert scale.

The participants were invited to participate in the online survey from the Turkish Crohn's and Ulcerative Colitis Patient Association network.

The primary outcome of our study was to determine the burden of IBD-related health problems at work and in social life and education in the study cohort. All programs were performed using the SPSS 21.0 package program. Frequency and percentages of categorical variables and mean and standard deviation or median and minimum-maximum values of continuous variables were calculated as descriptive statistics. The relationship between categorical variables was tested by the chi-square test or Fisher's precision test, and the relationship between continuous variables was tested by Spearman correlation analysis. The confidence level of the study was 95% (*p* < 0.05 was considered statistically significant).

## 3. Results

One hundred eighty IBD patients volunteered to participate; 79 were female (42.5%) and 107 were male (57.5%) (Table 1). One hundred and fifteen patients had UC (57.2%), and 86 had CD (42.8%). Most of the participants were clustered in age group 2 (between the ages of 30 and 39). 40.9% of the UC patients and 45.3% of the CD patients were in this age group (*p* > 0.05). 38.9% patients were not working (retired or unemployed).

Twenty-nine UC patients and 26 CD patients replied to the questions about retirement. Only 3 patients in each group claimed that they retired after the age of 65. The most prevalent retirement age interval was between the ages of 40 and 49 in both diseases, which is a pretty early age interval compared to that of healthy employers in Turkey.

There was a statistically significant difference in UC between retirement age group 1 (<40 age) and groups 2 (40-49 ages) and 4 (60-65 ages) (*p* < 0.05). There was the same significant difference between the groups 1 and 5, getting retired more in the first group (*p* < 0.05). Retirement age in CD was clustered between the ages of 30 and 39 (7 patients) (*p* < 0.023).

Disability due to IBD was perceived in 73 UC patients (63.5%), and the clustering was in age group 2 (30-39). In this age group, around 60% of them had the feeling of being disabled. The same clustering was determined in 20 CD patients (50.4%) in this age group. However, there was no statistically significant difference between the age groups.

The rate of feeling worried for unemployment was quite the same in both diseases and all age groups (*p* > 0.05).

The rate and the duration of absenteeism from work were similar in all groups (*p* > 0.05). The overall presenteeism rate was 29.1% and did not differ between the groups.

There was no difference between the global health scores patients gave themselves, 10 being the best and highest score between all the groups (*p* = 0.216).

Among 199 respondents, 6.5% gave 10 points (the very best) for their health status and 40.7% gave 10 points if they suppose they did not have IBD. 18% and 3% were the ratios of the patients who gave the worst point [3] for the corresponding questions.

**Figure 1:**
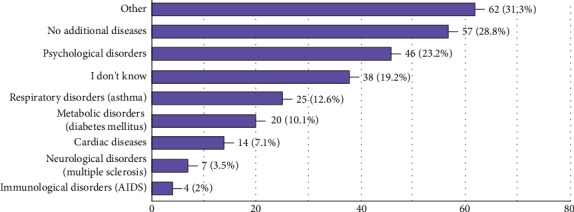
Additional chronic diseases the study population has.

**Figure 2:**
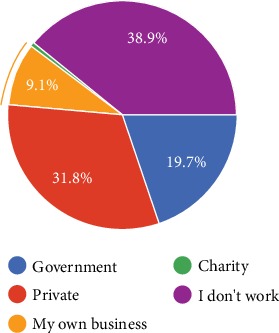
Type of employment the study population has.

The most prevalent pronounced additional disorder (Figure 1) was psychological disorder, and this data supports the fact that the chronicity of the disease has a huge psychological burden. Around 40% of the respondents (Figure 2) do not work even though most of the subjects were in the productive phase of their lives. Only 6 responders were 60 years old or older, and 48 responders were in the 16-29 age interval. If fifty-four patients were assumed not working due to senility or ongoing education, still about 25% of the patients can be presumed to be not working even though they were in the active work-productive phase. The unemployment rate is declared as 13% (half of the unemployment rate in IBD) in Turkey by the Turkish Statistical Institute in April 2019.

**Figure 3:**
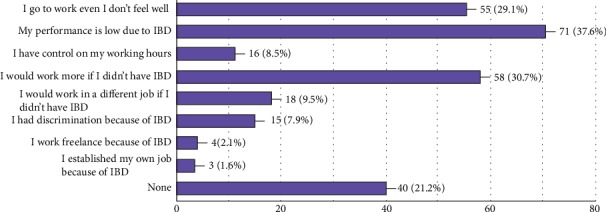
The belief patterns of the IBD patients about work.

Among the 189 (Figure 3) responders to the question about the impression or feelings about their working life (both the still-working and unemployed population), 149 of them had negative perceptions; among them, three leading subgroups admitted that they would still work at the same work; however, they thought that they would work more qualitatively and quantitatively if they did not have IBD.

**Figure 4:**
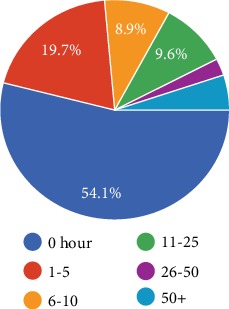
How many hours did you miss work in the last 7 days of work?

**Figure 5:**
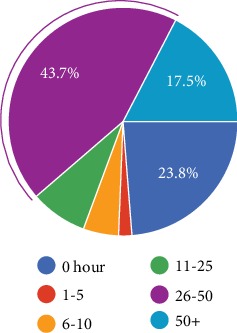
How many hours did you work in the last 7 working days?

More than half of the responders admitted that they did not go to work more than 50 hours in the last seven days of work (Figure 4). However, the replies to the consecutive questions were discrepant since there were only 23.8% replies as not working at all in the last seven working days (Figure 5). On the other hand, the discrepancy might have been caused by different types of work and working hours of each individual. For instance, some patients' total daily working hours were 3 hours while others' were 8 hours.

There were 2 questions interfering with the education life. Participants gave responses according to their past experiences or the difficulties they live concurrently. 40% totally agreed that IBD blocked their education life in some way. 37.4% admitted that they had lower potentials in their scholarships due to IBD.

## 4. Discussion

This prospective cohort study apparently shows that the burden of IBD is high in the Turkish population. About 50% of the patients reported loss of work productivity in the past 7 working days. IBD seems to affect career choice and also decision to retire. The retired respondents admit IBD had played some part in their decision to retire. The data indicated that there was no notable difference between the retirement ages of people with Crohn's and UC. Retirement ages are clustered in the very early ages in both UC and CD. The questionnaire also showed that IBD causes unemployment producing issues for the patients to work at all or find a suitable job.

It seems as if most of the patients had additional diseases; however, the debilitating diseases such as AIDS or cardiac disease are much less than the psychological disorders, and psychological disorders might be interpreted as a consequence of the chronic nature of IBD. Due to high percentage of additional diseases, it can be thought that work productivity can be affected by those additional diseases; however, there were two questions in the questionnaire which make the situation clear. The patients were asked “what score do you give to your health status for the last month?” and “what score would you give if you did not have IBD?” The scores were much higher to the second question making IBD the center problem in their lives.

Disability perception due to IBD was the most prevalent between the ages of 30 and 39 in both UC and CD. However, there was no statistically significant difference between the age groups. The rate and the duration of absenteeism from work were similar in all groups (*p* > 0.05). The overall presenteeism rate was 29.1% and did not differ between the groups. The influence of IBD on employment expectations in young people aged 16-29 is high. The survey affirmed IBD can have an effect on the educational attainment of young people. Around 38% of young participants stated that their IBD has hindered them from achieving their full potential.

Leong et al. had an unemployment rate of 40% in their CD cohort similar to the ACCENT 1 cohort of refractory CD patients' rate of 39% [13]. In this study, IBD-DI (IBD Disability Index) was validated and found out to be sensitive enough to detect work absenteeism of a mean of 1.3 h in the previous week.

Two studies that evaluated early retirement reported that 4% of CD and 5% of UC patients [14] and 7% of IBD patients [15] had early retirements [16]. Boonen et al. had declared employment was lower in patients compared to controls [17]. The difference was more noticeable in IBD patients with symptoms when compared to the ones without symptoms. The IBD cases with symptoms were more likely to be out of the labor force (OR = 2.14) [18]. Schwartz et al. reported a cohort comprised of 597 CD patients. 29.1% of the patients reported missing from work in the last week because of their disease, and they concluded that a long-term assessment of the effect of new pharmaceutical treatments on patients' productivity is warranted [19].

Our survey revealed a very strong causative relationship between work and IBD involving problems before, during, and at the end of employment. Young patients lower their career expectations, and that announces a clear need to support them and improve career guidance. Very basic adjustments in the workplace by the employer such as easy access to toilets or time for doctor/hospital appointments separate from holiday allowance could prevent absenteeism and presenteeism. To play their part in providing awareness of Crohn's and UC in the workplace, the patients should be in contact with each other and their national patient association. However, only 34% of the responders claimed that they were members of the patient association.

## Figures and Tables

**Table 1:** Gender, type of disease, age groups and retirement age groups, and disability perception.

	UC (*n* = 115)	CD (*n* = 86)	*p*
Sex (*n*, %)			
Male	48 (44.4%)	31 (39.7%)	>0.05
Female	60 (55.6%)	47 (60.3%)	
Age groups			
Group 1 (16-29)	25 (21.7%)	23 (26.7%)	
Group 2 (30-39)	47 (40.9%)	39 (45.3%)	
Group 3 (40-49)	27 (23.5%)	16 (18.6%)	>0.05
Group 4 (50-59)	11 (9.6%)	7 (8.1%)	
Group 5 (60 and above)	5 (4.3%)	1 (1.2%)	
Retirement age groups			
Group 1 (<40)	5 (17.2%)	9 (34.6%)	
Group 2 (40-49)	11 (37.6%)	9 (34.6%)	
Group 3 (50-59)	8 (27.6%)	3 (11.5%)	>0.05
Group 4 (60-65)	2 (16.9%)	2 (7.7%)	
Group 5 (66 and above)	3 (10.3%)	3 (11.5%)	
Disability perception			
Yes	73 (63.5%)	49 (58.3%)	>0.05
No	42 (36.5%)	35 (41.7%)	

## Data Availability

Data will be provided by the corresponding author in case of any request.
